# Effects of the Proteasome Inhibitor Bortezomib in Combination with Chemotherapy for the Treatment of Mantle Cell Lymphoma: A Meta-analysis

**DOI:** 10.4274/tjh.galenos.2019.2019.0128

**Published:** 2020-02-20

**Authors:** Shi-Jun Li, Jian Hao, Yu Mao, Yu-Ling Si

**Affiliations:** 1 Tianjin 4th Central Hospital, Clinic of Hematology, Tianjin, China; 2 Renmin Hospital, Clinic of Oncology, Tianjin, China

**Keywords:** Bortezomib, Chemotherapy, Mantle cell lymphoma, Metaanalysis

## Abstract

**Objective:**

The efficacy and the safety of bortezomib-based chemotherapy were characterized in mantle cell lymphoma (MCL) patients.

**Materials and Methods:**

The PubMed, Cochrane Library, Clinical Key, Science Direct, Oxford Journals, and China National Knowledge Internet databases were searched up to 1 May 2019. The selected trials needed to match the inclusion criteria and be carried out to evaluate quality appraisal and the synthesis of efficacy and safety. The enrolled MCL patients using bortezomib-based chemotherapy or chemotherapy alone needed to have been compared. The overall response rate (ORR), progression-free survival (PFS), and overall survival (OS) were combined to evaluate the efficacy while serious adverse events (SAEs) (grade III-IV peripheral neuropathy, neutropenia, and infection) were used to evaluate the safety. The heterogeneity of the results were analyzed simultaneously.

**Results:**

A total of 620 patients were enrolled across four studies in our meta-analysis, and the pooled results showed that the PFS [hazard ratio (HR)=0.66, 95% confidence interval (CI)=0.54-0.82; p=0.0001)] and OS (HR=0.73, 95% CI=0.55-0.96; p=0.03) of patients with bortezomib-based chemotherapy were better than those of patients with chemotherapy alone, unlike ORR (risk ratio=1.46, 95% CI=0.85-2.49; p=0.17), while SAEs were prominent in the combination group.

**Conclusion:**

MCL patients who are ineligible for transplant or high-dose chemotherapy could benefit from bortezomib-based chemotherapy.

## Abstract (tr)

**Amaç:**

Mantle hücreli lenfoma (MCL) hastalarında Bortezomib bazlı kemoterapinin etkinliği ve güvenilirliğinin belirlenmesi.

**Gereç ve Yöntemler:**

1 Mayıs 2019 tarihine kadar PubMed, Cochrane Kütüphanesi, Klinik Anahtar, Doğrudan Bilim, Oxford Dergileri ve Çin Ulusal Bilgi İnternet veritabanları araştırıldı. Seçilen çalışmaların dahil edilme kriterlerini karşılaması ve kalite, ve etkinlik ve güvenlik sentezi yapmış olması gerekiyordu. Çalışmaların bortezomib bazlı kemoterapi ile sadece kemoterapi kullanan MCL hastalarını karşılıyor olması gerekiyordu. Etkinliği değerlendirmek için genel yanıt oranı (ORR), ilerlemesiz sağkalım (PFS) ve genel sağkalım (OS) birleştirilirken, ciddi advers olaylar (SAE’ler) (3-4. düzey periferik nöropati, nötropeni ve enfeksiyon) güvenliği değerlendirmek için kullanıldı. Sonuçların heterojenliği aynı anda analiz edildi.

**Bulgular:**

Meta-analizimizde dört çalışmaya toplam 620 hasta dahil edilmişti ve toplu sonuçlarda PFS [tehlike oranı (HR)=0,66, %95 güven aralığı (CI)=0,54-0,82; p=0,0001)] ve OS (HR=0,73, %95 CI=0,55-0,96; p=0,03) ORR’den (risk oranı=1,46, %95 CI=0,85-2,49; p=0,17) farklı olarak bortezomib bazlı kemoterapi alan hastalarda, tek başına kemoterapi alan hastalardan daha iyi idi, SAE’ler ise kombinasyon grubunda daha belirgindi.

**Sonuç:**

Nakil veya yüksek doz kemoterapi için uygun olmayan MCL hastaları bortezomib bazlı kemoterapiden yarar görebilir.

## Introduction

Generally, mantle cell lymphoma (MCL) is an aggressive, incurable subtype of non-Hodgkin B cell lymphoma [1,2,3], with cyclin D1 overexpression resulting from t(11;14) (q13;q32) translocation [4,5]. High-dose chemotherapy with or without consolidation followed by autologous hematopoietic stem cell transplantation (ASCT) is the first-line treatment for MCL patient [2]. For patients not suitable for high-dose chemotherapy or transplant, reduced-dose chemotherapy is recommended [1,2,4]. However, there are no generally accepted therapeutic approaches to date. Combined chemotherapy regimens like cyclophosphamide, doxorubicin, vincristine, prednisone, and rituximab (R-CHOP) or rituximab, hyperfractionated cyclophosphamide, vincristine, doxorubicin, and dexamethasone (hyper-CVAD), and/or high-dose consolidation therapies, are frequently used. However, the median failure-free survival for standard therapy is only 8 to 20 months and the median survival of patients with high-intensity chemotherapy is 3-4 years [6]. A number of novel agents were later approved for MCL, including bortezomib, lenalidomide, and ibrutinib. Among them, ibrutinib obtained the most significant effects with over 60% overall response rate (ORR) and almost 20% complete remission (CR) in relapsed/refractory (R/R) MCL [7], but it is not widely available for patients in developing countries with expensive costs. Lenalidomide did not benefit MCL patients with the minimum ORR and CR in R/R MCL [8].

Bortezomib was confirmed to have a durable response and a favorable rate of progression-free survival (PFS) in single-agent data for R/R MCL in a multicenter phase II study [9], which contributed to it being approved by the FDA for the treatment of MCL patients in relapse after prior therapy. The SWOG S0601 trial further showed that the combination of bortezomib with R-CHOP followed by bortezomib maintenance obtained a doubled 2-year PFS rate compared with the R-CHOP regimen alone (62% vs. 30%) in previously untreated MCL patients [10]. However, a randomized phase II study assessed the efficacy of bortezomib plus CHOP versus CHOP in relapsed MCL patients and showed that bortezomib-based chemotherapy had a non-significant improvement on PFS (16.5 months vs. 8.1 months; p=0.12) [11]. To obtain a better understanding of bortezomib combination therapy in MCL patients, we performed a meta-analysis of clinical trials to compare the efficacy and safety of bortezomib-based chemotherapy in MCL patients.

## Materials and Methods

### Literature Sources

A literature review was performed by two reviewers independently on the efficacy and safety of bortezomib-based chemotherapy for MCL patients in the PubMed, Cochrane Library, Clinical Key, Science Direct, Oxford Journals, and China National Knowledge Infrastructure databases in both English and Chinese. All relevant studies reported up to 1 May 2019 were searched and the search terms included “mantle-cell lymphoma” or “MCL” and “bortezomib” or “Velcade” alone or together. In addition, the published reference lists of those articles were also checked for further eligible publications.

### Inclusion Criteria

The eligible studies needed to conform to the following inclusion criteria: (1) the trials enrolled MCL patients who were newly diagnosed, previously untreated, in first CR, or relapsed; (2) the trials included randomized controlled trials (RCTs) or prospective cohort trials with a coincident or historical control group; (3) the trials provided sufficient data on bortezomib-based chemotherapy for MCL patients, including the hazard ratio (HR) of the overall survival (OS) and the PFS or the odds ratio (OR) of the clinical-pathological factors, which could be calculated along with the corresponding 95% confidence interval (CI); (4) if data were presented in more than one article, the most recent or the most elaborate study would be selected; (5) reviews, case reports, editorial comments, or letters to the editor without original data were not included.

### Data Collection and Quality Assessment

All titles and abstracts were screened by two reviewers independently. Disagreements between the two reviewers were settled by discussions to reevaluate the methodological quality of original studies. The Jadad scale was used to evaluate the methodological quality of the included RCTs, ranging from 0 to 7 points [12]. A high-quality study would have a score of 4 or greater. The Newcastle-Ottawa Quality Assessment Scale (NOS) was used to evaluate the quality of the cohort trials with a coincident or historical control group, ranging from 0 to 9 points. More than 5 points could be regarded as high quality.

### Outcome Calculation

Full extraction was performed on the comparative studies, including RCTs and cohort trials with a coincident or historical control group. The ORR, PFS, and OS were evaluated for efficacy. Serious adverse events [(SAEs); grade III/IV peripheral neuropathy, neutropenia, and infection)] were evaluated for safety. Adverse events were classified in terms of each individual clinical trial.

### Statistical Analysis

RevMan version 5.2 was used to perform all calculations related to the meta-analysis. Dichotomous data (ORR, peripheral neuropathy, neutropenia, and infection) were calculated in terms of a fixed or random effect model and expressed by the risk ratio (RR) or OR with 95% CI. Time-to-event results were expressed by HR and 95% CI and pooled with an inverse variance method through a fixed effect model. Because ORR is not a minor probability event, it was usually expressed as RR. Adverse events were generally expressed as OR. The inconsistency index (I^2^) and the χ^2^-based Cochran Q statistic were applied for heterogeneity detection between clinical trials. In terms of the values of the heterogeneity test, different analysis models were chosen: if I^2^>50%, a random effect model would be needed; in contrast, when I^2^≤50%, a fixed effect model would be selected. When assessing the difference in outcome, heterogeneity involving all trials was examined. A value of p<0.05 was considered statistically significant.

## Results

### Clinical Trials

We identified 2201 records in a primary literature search. After removing 1719 studies that included review articles, case reports, commentaries, single-arm trials, and phase I trials, 482 articles were identified for review. Then, after excluding duplicate or redundant studies and those lacking original data, only 4 eligible studies met the inclusion criteria of this meta-analysis [11,13,14,15]. All included clinical trials were presented as full publications; the characteristics of these trials are summarized in Table 1, including the name of the first author, year of publication, country, study design, detailed information on patients, therapy regimens, median follow-up time, PFS, OS, and quality score. As labeled in Table 1, Furtado et al. [11], Robak et al. [13], and Wu et al. [14] were RCTs, and William et al. [15] was a prospective cohort trial. All included clinical trials were determined to be of high quality.

### Overall Response Rate, Progression-free Survival, and Overall Survival

**Figure 1:**
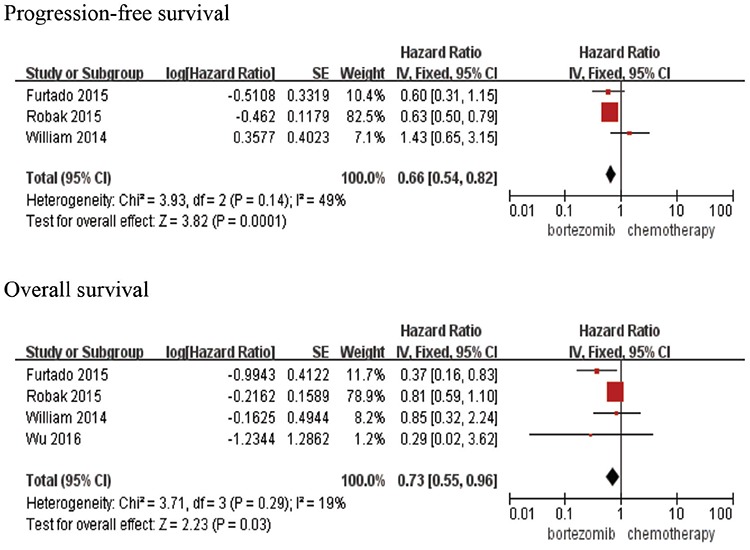
Pooled analyses of progression-free survival and overall survival. CI: Confidence interval.

The efficacy of bortezomib-based therapy could be confirmed by ORR and survival analysis in the above clinical trials (Table 1). The pooled RR for ORR was 1.46 (95% CI=0.85-2.49; p=0.17). There was no significant difference between bortezomib-based chemotherapy and chemotherapy alone in terms of ORR. Bortezomib-based chemotherapy had distinctly longer PFS (HR=0.66, 95% CI=0.54-0.82; p=0.0001) and OS (HR=0.73, 95% CI=0.55-0.96; p=0.03) (Figure 1) than chemotherapy alone in MCL patients.

### Serious Adverse Events

Three studies reported SAEs, including grade III/IV peripheral neuropathy, grade III/IV neutropenia, and grade III/IV infection [11,13,14]. The pooled OR for grade III/IV peripheral neuropathy, grade III/IV neutropenia, and grade III/IV infection was 2.44 (95% CI=1.02-5.83; p=0.04), 2.73 (95% CI=1.80-4.13; p<0.00001), and 1.83 (95% CI=1.15-2.92; p=0.01) respectively. SAEs were increased significantly in combination therapy compared with chemotherapy alone.

### Heterogeneity and Sensitivity Analysis

The heterogeneity of ORR was significantly different among the 4 pooled trials (χ^2^=12.72; df=2; I^2^=84%; p=0.002). The heterogeneity of grade III/IV peripheral neuropathy (χ^2^=0.74; df=2; I^2^=0%; p=0.69), grade III/IV neutropenia (χ^2^=0.66; df=2; I^2^=0%; p=0.72), and grade III/IV infection (χ^2^=1.11; df=2; I^2^=0%; p=0.57) exhibited a non-significant difference among the four pooled trials.

## Discussion

MCL is an incurable aggressive B-cell lymphoma with poor prognosis. The better treatment choice for MCL patients is high-dose chemotherapy containing cytarabine, followed by ASCT [16,17]. For patients who are either ineligible or not considered for intensive chemotherapy and ASCT, the standard R-CHOP regimen followed by rituximab maintenance is most commonly used [18], which could improve response duration compared with currently available therapies [19], but relapse is inevitable. A number of novel agents have been approved in the treatment of MCL, including bortezomib, lenalidomide, and ibrutinib. Among them, ibrutinib, a first-generation BTK inhibitor, obtained the most significant effects with over 60% ORR and almost 20% CR in R/R MCL [7]. On the contrary, lenalidomide obtained the minimum ORR and CR in R/R MCL [8]. Although ibrutinib has changed the landscape of therapy for MCL, it needs continuous administration until disease progression or unacceptable drug-related toxicity. That will be expensive and it is not widely available for patients in developing countries, especially in China. In addition, most of the patients receiving ibrutinib experienced common adverse events, including diarrhea (54%), fatigue (50%), bleeding (50%), nausea (33%), cytopenias (20%), atrial fibrillation (11%), dyspnea (32%), and pneumonitis (8%) [7,20], inevitably leading to the discontinuation of therapy. More recently, acalabrutinib, a second-generation BTK inhibitor, has demonstrated promising efficacy with 81% ORR and 40% CR for R/R MCL in a phase II study along with lower rate of toxicities[21]. However, it still needs continuous administration until disease progression or unacceptable drug-related toxicity, which would be unacceptable for most patients.

Bortezomib, the first proteasome inhibitor, regulates multiple cell signaling pathways related to the progress of MCL. It can reversibly depress the 26S proteasome for inhibition of nuclear factor-κB and TP53, and it can induce cell cycle arrest and apoptosis [22]. Bortezomib was approved by the FDA for the treatment of MCL patients in relapse after it was confirmed to have 31% OS and median response duration of 9.3 months in single-agent activity for R/R MCL [9]. Afterwards, bortezomib obtained significant prolongation of PFS and OS in newly diagnosed MCL patients when combined with standard chemotherapy in a phase 3 clinical trial [13]. Based on these studies, bortezomib was approved in the USA and Europe for the treatment of MCL patients in both relapsed and upfront settings [23,24]. Furthermore, bortezomib is low-cost and easy to obtain. However, in a randomized phase II study, there was a non-significant improvement in PFS (16.5 months vs. 8.1 months; p=0.12) when assessing the efficacy of bortezomib-CHOP (cyclophosphamide, doxorubicin, vincristine, and prednisone) versus CHOP in relapsed MCL patients [11]. We thus collected bortezomib-based clinical trials to explain the efficacy and safety of bortezomib-based regimens.

In this meta-analysis, four studies were included [11,13,14,15]. There were no significant differences in baseline characteristics between the treatment group and control group in each clinical trial. Among them, three studies proved the benefits of bortezomib regimens in MCL patients. The Furtado et al. [11] and Wu et al. [14] studies showed the efficacy and toxicity of a CHOP-bortezomib regimen compared with the CHOP regimen in MCL patients at first relapse. In Furtado et al. [11], a marked improvement in the quality of responses was achieved when bortezomib was added to CHOP chemotherapy, with 82.6% vs. 47.8% of patients obtaining an objective response (CHOP-bortezomib vs. CHOP, respectively). The OS in the CHOP-bortezomib arm was 35.6 months compared with 11.8 months in the CHOP arm, but there was no difference in PFS between the CHOP-bortezomib arm (16.5 months) and CHOP arm (8.1 months), although 30.4% of patients progressed during treatment in the CHOP arm compared to 8.7% in the CHOP-bortezomib arm [11]. Moreover, there were slightly more patients experiencing additional toxicities attributed to the inclusion of bortezomib. Wu et al. [14] also proved the benefits of a CHOP-bortezomib regimen for ORR and OS without increasing adverse events. The ORR of CHOP-bortezomib was higher than that of the CHOP arm (84.2% vs. 42.1%), and the median OS of the CHOP-bortezomib arm was 56.0 months, which was longer than the 29.0 months of the CHOP arm. Robak et al. [13] compared the efficacy and toxicity between newly diagnosed MCL patients who received R-CHOP and VR-CAP (bortezomib, rituximab, cyclophosphamide, doxorubicin, and prednisone). The results showed that the VR-CAP group had a significant improvement in PFS but no significant improvement in OS and ORR. Because the median OS was not reached in the VR-CAP arm at the time of the study, there was only a non-significant improvement in OS with an improved 4-year OS rate compared with the R-CHOP arm (64% vs. 54%). Compared with the R-CHOP group, the patients receiving VR-CAP treatment also had more adverse events, which were mainly neutropenia and thrombocytopenia. In addition, William et al. [15] compared the OS and PFS between a BEAM (carmustine, etoposide, cytarabine, and melphalan)-bortezomib regimen and a BEAM regimen in MCL patients who were evaluated as CR1. The results showed that patients receiving the BEAM-bortezomib regimen had non-significant improvement in OS and PFS compared with the BEAM regimen. In that study, the patients enrolled were in the first CR after receiving upfront therapy with a rituximab and hyper-CVAD regimen, and they received ASCT after a BEAM-bortezomib/BEAM regimen treatment. Although there were no significant improvements in OS and PFS, the authors considered the benefit of bortezomib to have not been revealed in the presence of ASCT; in other words, bortezomib could offer benefits for MCL patients who are ineligible for ASCT. After all, ASCT benefits only about 60% of MCL patients.

The reported ORR, OS, PFS, and SAE values were pooled from the above four trials. The meta-analysis showed that patients who received bortezomib-based therapy had longer PFS and OS compared with those receiving chemotherapy alone, but there was no significant difference in ORR. The reasons might be as follows: first, the pooled data on ORR were extracted from three studies (Furtado et al. [11], Robak et al. [13], and Wu et al. [14]), among which Robak et al. [13] enrolled the most patients and held the highest weight; however, Robak et al. [13] showed that the bortezomib-based group had no significant improvement in ORR, which might have influenced the heterogeneity of ORR. Thus, more research should be conducted to reevaluate the pooled ORR. Second, Robak et al. [13] showed that the VR-CAP arm had a non-significant improvement in OS with an improved 4-year OS rate compared with the R-CHOP arm (64% vs. 54%); meanwhile, 132 patients (54%) in the R-CHOP arm and 82 patients (34%) in the VR-CAP group received subsequent anti-lymphoma therapy, and the type of subsequent therapy was generally similar in the two groups. However, the other three studies did not mention subsequent anti-lymphoma therapy after disease progression, and subsequent anti-lymphoma therapy might influence OS. Despite the advantages, our results suggest that a bortezomib-based regimen might cause significant increases in SAEs (i.e. grade III/IV peripheral neuropathy, infection, and neutropenia).

In addition, the post hoc sub-analysis of Robak et al. [13] assessed the efficacy and safety of VR-CAP and R-CHOP in 80 MCL patients aged <60 years who did not receive stem cell transplantation despite medical eligibility [25]: the median PFS and the median OS in the two groups were 42.6 vs. 20.6 months (HR=0.54; p=0.057) and not reached vs. 47.3 months (HR=0.81; p=0.634), which suggested that VR-CAP had superior efficacy to R-CHOP in suitable young MCL patients. Another sub-group analysis of Robak et al. [13] investigated whether VR-CAP compared with R-CHOP could improve outcomes in East Asian patients with newly diagnosed MCL [26]: the results supported the benefits of VR-CAP in East Asian patients with MCL who are ineligible for transplant, as the median PFS was 27.7 months (VR-CAP) vs. 16.1 months (R-CHOP) (HR=0.58; p=0.03), and the median OS was not reached (VR-CAP) vs. 56.3 months (R-CHOP).

This meta-analysis has some limitations. First of all, because a great mass of phase 3 RCTs on bortezomib are not yet finished, only three RCTs were included in our study, and this was the main limitation of the meta-analysis. Second, some articles were short of data on CR and the other common SAEs such as thrombotic events, so we could not compare the outcomes of the two regimens. Third, the clinical stage of the patients and the selection of combined chemotherapies were disparate among all trials, which would bring about heterogeneity. In the future, more phase 3 RCTs concerning bortezomib regimens with or without other new medicines could help to formulate a conclusion regarding MCL treatment; they may offer better efficacy and fewer adverse events. Despite the limitations mentioned above, we have affirmed that bortezomib-based regimens make a valuable contribution to the treatment of MCL patients who are either ineligible or not considered for intensive chemotherapy and ASCT.

## Conclusion

In summary, bortezomib-based regimens for MCL patients were more effective than chemotherapy alone in our analysis, but with more grade III/IV adverse events. Bortezomib-based therapy is more suitable for MCL patients who are either ineligible or not considered for intensive chemotherapy and ASCT.

## Figures and Tables

**Table 1:**
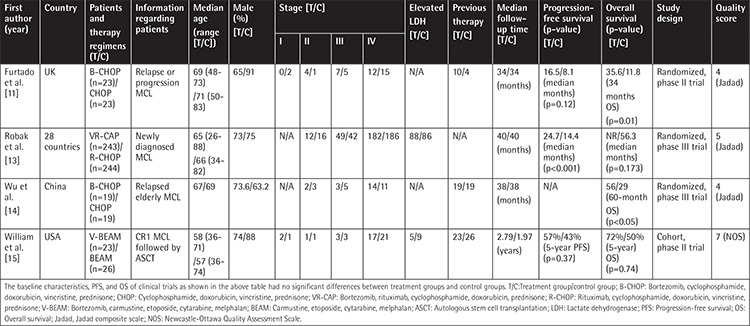
Comparison among 4 trials for baseline characteristics, PFS, and OS.
